# State Controlled and Voluntary Hospitals

**Published:** 1919-06-07

**Authors:** 


### STATE CONTROLLED AND VOLUNTARY HOSPITALS.

The great difference in the past between the State-controlled and the voluntary hospital has been that in the first the patients possess few, if any, rights or protection, whereas, as we have pointed out, under the latter or voluntary system the patient comes first, and properly first, all the time. The one grave danger which threatens the voluntary hospital system at the moment in this country is the general and widespread ignorance which prevails of the actual conditions of State-controlled hospitals, what such conditions have meant really in practice, and the kind of service they have extended to their patients. It is time that this ignorance should be dispelled, and that the underlying principles and splendid service the best of our voluntary hospitals give to their patients should be made plain. British men and women need the knowledge that we can only have a Merrie England for sick and injured people where the voluntary system prevails and is paramount. For then the patients' rights, treatment, and comfort will be everywhere the primary principles. This is not the case under a hospital system which is State-controlled from top to bottom.

There are many politicians whose utterances may be eloquent, but whose ignorance is profound, that fulminate against the voluntary hospitals, and extol the merits and glories of a State system, not only of hospitals but of everything. It is lamentable indeed, and says little for a system of education which has been allowed to degenerate so seriously in this country, that the ignorance of the bulk of the people, and of their would-be leaders and masters, is demonstrably profound. We need to secure the dissemination of truth in regard to the only basis on which the defenceless sick can be guaranteed, as the first consideration, the best of care, the tenderest, most skilled and adequate treatment, and the presence of everything which can promote the speediest possible recovery in public institutions where sick and injured people are admitted to treatment. The voluntary hospitals owe it to their deservedly high reputation that steps should be taken to awaken the public to the true inwardness of their work, and the splendid results which have been achieved under their administration for British people of both sexes and all ranks, not forgetting the children. The sooner steps are taken to institute such a crusade the better will it be for the well-being and protection of the working and poorer classes throughout the United Kingdom.

The Treasurer of the Radcliffe Infirmary, the Rev. G. B. Cronshaw, of Queen's College, Oxford, has usefully drawn attention in the *Oxford Chronicle* to the difference between State-controlled and voluntary hospitals. He rightly affirms that these two systems—one State-controlled and one under voluntary management—have been working side by side for the past four years in the cast-iron discipline and service of the military hospitals, and in the elasticity and sympathy of the Red Cross auxiliary hospitals under voluntary management, and there is no doubt which of these two systems the country would wish to keep for always. We feel that Mr. Cronshaw is right, and that it is essential in the reorganisation of the British hospital system, which must come in the near future, that the public must be awakened to the paramount principles on which the voluntary hospital system rests. This system all hospital managers at the present time should do their very best to safeguard and uphold. Mr. Cronshaw fears disaster for the sick, and we affirm this must be the case if the greatest care is not taken that the interests of the patients who seek our help shall everywhere be upheld. To secure this end, every one of us who has knowledge and experience must combine, for then the loving care and interest which are now bestowed on our patients can always be theirs. It will need the effectual shutting of every British hospital's doors against the State's soullessness, to be found in institutions where the principle of the claims of the patient before all others does not prevail.

